# Cardiovascular Risk in Patients with Psoriatic Arthritis

**DOI:** 10.1155/2012/714321

**Published:** 2012-05-08

**Authors:** Tracy Y. Zhu, Edmund K. Li, Lai-Shan Tam

**Affiliations:** Department of Medicine and Therapeutics, Prince of Wales Hospital, The Chinese University of Hong Kong, Shatin, N.T., Hong Kong

## Abstract

Psoriatic arthritis (PsA) is an inflammatory arthritis associated with psoriasis. In addition to skin and joint involvement, there is increasing evidence suggesting that patients with PsA also have an increase in risk of clinical and subclinical cardiovascular diseases, mostly due to accelerating atherosclerosis. Both conventional and nonconventional cardiovascular risk factors contribute to the increased cardiovascular risk in PsA. Chronic inflammation plays a pivotal role in the pathogenesis of atherosclerosis in PsA, acting independently and/or synergistically with the conventional risk factors. In this paper, we discuss the current literature indicating that patients with PsA are at risk of cardiovascular diseases.

## 1. Introduction

Psoriatic arthritis (PsA) is an immune-mediated inflammatory arthritis associated with psoriasis. Annual incidence of PsA ranges from 0.1 to 23.1 per 100,000, while prevalence ranges from 1 to 420 per 100,000 [1]. Patients with PsA have heterogeneous clinical presentations with diverse articular and dermatological features as well as varied disease course and outcomes. PsA can be a severe form of arthritis with prognosis similar to that of rheumatoid arthritis (RA) [2–4]. In addition to skin and musculoskeletal involvement, there is increasing evidence suggesting that patients with PsA also have an increase in risk of cardiovascular disease (CVD) [5–7]. The objective of this paper is to provide an overview of the current literature indicating that patients with PsA are at risk of CVD. A better knowledge of the association between PsA and cardiovascular comorbidities can help in early management and modification of risk factors, minimize the impact of the cardiovascular comorbidities, and improve patients' long-term outcome. 

## 2. Literature Search

Literature search was conducted using the PubMed database up until January 2012. Key words for the search were “psoriatic arthritis” in combination with “mortality”, “cardiovascular disease”, “coronary heart disease”, “coronary artery disease”, “cardiovascular risk”, “atherosclerosis”, “subclinical atherosclerosis”, “endothelial function”, “lipids”, “hypertension”, “diabetes mellitus”, “homocysteine”, or “acute-phase proteins” in the title, abstract, or key words with limits set to include humans and studies written in English. Articles were deemed eligible if they included (cardiovascular) mortality and morbidity, and/or subclinical cardiovascular morbidity, and/or data about cardiovascular risk factors, and/or the effects of treatment on cardiovascular risk in PsA. The initial search yielded 528 abstracts, which were narrowed to 90 potentially relevant articles by preliminary review of the titles and by excluding review articles and case report (*n* = 126). Twenty articles were deemed ineligible after examining the abstracts. Full texts of the remaining 70 articles were retrieved. The reference lists of retained papers were then checked for any articles that the initial database search had failed to identify, yielding 10 additional articles for full text examination. The majority of articles excluded were due to only providing data on patients with psoriasis or due to being not relevant to the cardiovascular risk in PsA. In the end, 52 articles were deemed eligible for this paper.

The diagnostic criteria used in each included study are not uniform. PsA was diagnosed as (seronegative) inflammatory arthritis associated with psoriasis in 14 studies. Moll and Wright criteria for PsA and the ClASsification for Psoriatic ARthritis criteria were used in 9 and 10 studies, respectively. Diagnosis of PsA was obtained from patient charts or database in 6 studies. Thirteen studies did not provide clear diagnostic criteria.

## 3. Mortality and Cardiovascular Mortality in PsA

Results from the current mortality studies in PsA yield conflicting findings [3, 8–17] (Table 1). The study by Shbeeb et al. on a small community-based incident cohort (*n* = 66) of Minnestona, USA, found that the survival of patients with PsA was not significantly different from that of the general population [10]. A later study with a large sample (*n* = 147) over 3 decades by the same group confirmed this finding [9]. The study by Buckley et al. on a cohort of 453 patients derived mainly from primary care also concluded that mortality of PsA did not differ significantly from that of the general United Kingdom population [14]. However, excess mortality in patients with PsA was documented in several large studies [8, 12, 15, 17]. In a mortality study of 428 patients with PsA from the University of Toronto Psoriatic Arthritis Clinic with a mean follow-up of 11.4 years, the standardized mortality ratio (SMR) for the female cohort was 1.59, and for the men, it was 1.65, indicating a 59% and 65% increase, respectively, in the death rate compared with the general population [8]. A later analysis from the same cohort by Ali et al. showed improvement in mortality over three decades, particularly for male patients [15]. However, overall mortality still increased significantly in female patients and in the overall study cohort [15]. These conflicting findings may be partially explained by the characteristics of the cohorts. The studies reporting no increase in mortality in PsA are either having a small cohort [9, 10] or more likely to represent early and less severe stage of the disease in their cohorts [9, 14]. Since mortality among patients with PsA is related to disease severity [18, 19], excess mortality is more likely to occur in clinic-based cohorts with overrepresented severe PsA [15]. On the other hand, the 2 studies in Denmark [17] and Hong Kong [12], respectively, both using large nationwide registry database and probably representing a broad spectrum of the disease, confirmed increased mortality of PsA than that of the general population. The study in Denmark provided rate ratio for mortality compared with over four million controls derived from general population, adjusted for age, calendar year, concomitant medication, comorbidity, socioeconomic data, and gender [17]. Mortality in patients with PsA was found similar to that in patients with psoriasis only [17]. Adjusted SMRs were available in the study by Ali et al., where mortality rates were adjusted for radiological damage, erythrocyte sedimentation rate (ESR), presence of hypertension, the number of actively inflamed joint and smoking status at entry into the clinic [15]. Results were presented as the 10-year “rolling-average” SMRs. After adjustment, the decline in mortality over three decades remained evident in male patients, but the decline was less marked for the overall population and no decline was apparent for female patients [15].

 The overall picture of the current mortality studies suggests an increased in mortality among patients with PsA. The excess mortality is predominantly due to CVD. Majority of the previous studies found CVD as the leading cause of death, responsible for 20% [12] to 56% [3] of all causes of death (Table 1). Cardiovascular mortality risk ratio versus the general population was available in the study by Wong et al. [8]. Cardiovascular mortality in PsA was found 30% higher than that in the general population [8]. Deaths due to CVD were divided into myocardial infarction (28%), cerebrovascular accidents (4%), and congestive heart failure/atherosclerosis (4%) [8].

## 4. Cardiovascular Morbidity in PsA

Data are limited regarding cardiovascular morbidity in patients with PsA [20–24]. Han et al. investigated the prevalence of CVD in 3,066 patients with PsA compared to 12,264 healthy controls in a cross-sectional cohort [22]. The age- and sex-adjusted prevalence of chronic heart failure (1.9 versus 1.3), ischemic heart disease (7.3 versus 5.5), peripheral vascular disease (2.9 versus 1.9), cerebrovascular disease (3.1 versus 2.3), type II diabetes (11.3 versus 7.3), hyperlipidemia (27.8 versus 23.7), and hypertension (28.5 versus 22.3) was significantly higher in patients with PsA than that in healthy controls, indicating an increased risk of cardiovascular morbidity. Gladman et al. conducted a prospective study on prevalence of cardiovascular morbidity on 648 patients of PsA with a mean follow-up of 8.3 years at the University of Toronto Psoriatic Arthritic Clinic [23]. Two hundred and twenty-seven patients had at least one cardiovascular condition (hypertension, cerebrovascular accident, myocardial infarction, angina, or congestive heart failure), corresponding to a prevalence of 35%. Increased prevalence was observed for hypertension, myocardial infarction, and angina with overall age and sex-adjusted standardized prevalence ratios of 1.9, 2.6, and 2.0, respectively. The prevalence of cerebrovascular accident and congestive heart failure was not found significantly increased. Two cross-sectional studies found mixed association between PsA and cardiovascular morbidity [20, 21]. Patients with PsA were not found to have increased prevalence of CVD [20, 21] or diabetes mellitus [20] but had increased prevalence of hypertension [20, 21] and heart failure [20]. However, results from these two studies should be interpreted with caution in view of the relatively small samples (99 [20] and 165 [21] patients, resp.) and the reliance on patient-reported information [21]. 

 In summary, there is an increased prevalence of cardiovascular morbidity and their risk factors in patients with PsA when compared with general population. The prevalence of CVD (including myocardial infarction, stroke, and/or transient ischemic attack) in PsA (10%) was found to resemble that in RA (12%) in a cross-sectional study on 489 patients with PsA and 353 patients with RA [25]. The age- and sex-adjusted odds risk of CVD showed no significant difference between patients with RA and those with PsA [25]. In a recent cross-sectional study by Husted et al., prevalence of cardiovascular morbidities was compared between 611 patients with PsA and 449 patients with psoriasis only [24]. Results indicated significantly increased prevalence of hypertension (37.1% versus 19.6%), obesity (30% versus 26.5%), hyperlipidemia (20.7% versus 14.5%), type 2 diabetes mellitus (12% versus 6.7%), and at least 1 cardiovascular event (8.2% versus 3.3%, including angina, myocardial infarction, cardiomyopathy, congestive heart failure, and cerebrovascular accident) among patients with PsA compared with those with psoriasis only. The increased prevalence of hypertension remained significant after adjusted for demographics, psoriasis severity, and use of medication. Overall, these findings tentatively support the role of inflammatory arthritis in cardiovascular morbidity in patients with PsA.

## 5. Subclinical CVD in PsA

There are several literatures identifying subclinical CVD which precedes overt cardiovascular events and mortality in patients with PsA. Results support that subclinical atherosclerosis is accelerated in PsA.

Endothelial dysfunction is considered an early feature in atherogenesis and has been consistently associated with cardiovascular risk [26]. It encompasses an imbalance between vasodilating and vasoconstricting substances, leading to an impaired ability of the artery to dilate in response to physical and chemical stimuli [26]. Postocclusion flow-mediated vasodilatation (FMD%) of the brachial artery using ultrasonography is used to noninvasively evaluate endothelial function [27]. In the study by Gonzalez-Juanatey et al. in 50 patients with PsA without cardiovascular risk factors or clinically evident CVD, FMD% was found significantly lower in patients with PsA compared with 50 matched healthy controls, indicating endothelial dysfunction in PsA as a potential basis for the association between PsA and atherosclerosis [28]. 

 Intima-media thickness (IMT) of the common carotid artery, determined by ultrasonography, is a valuable noninvasive surrogate marker of macrovascular atherosclerosis disease [29]. IMT corresponds to the width of the vessel intima and media, which is the site of lipid deposition and plaque formation [30]. Increased carotid IMT is a good indicator of generalized atherosclerosis and coronary artery disease, providing early information on atherosclerosis in subclinical stages of the disease [30]. All previous case-control studies, except 1 study involving a small sample [31], have demonstrated that patients with PsA have a higher prevalence of subclinical arthrosclerosis (Table 2). The largest increase (23%) in carotid IMT in PsA was found in a study by Tam et al. in a Chinese cohort [32]. The smallest increase in carotid IMT (8.7%) was found in the study by Gonzalez-Juanatey et al. in patients with PsA without cardiovascular risk factors or clinically evident CVD, significantly higher than that in matched healthy controls [33]. The prevalence of plaques in patients with PsA was also significantly increased compared with the healthy controls [32]. There was a significant linear trend of more severe plaques among patients with PsA [34]. The increase in the prevalence of plaque was not significant in patients with PsA without cardiovascular risk factors or clinically evident CVD; however, patients with PsA might be susceptible to more unstable atherosclerotic plaques [33]. 

Arterial stiffness is independent predictors of the risk of future fatal and nonfatal cardiovascular events and total mortality in various patients group and may also directly accelerate the atherosclerotic process [35]. The pulse wave velocity (PWV) is determined by the elasticity and other properties of the artery which correlate with arterial distensibility and stiffness. An increase in aortic PWV by 1 SD corresponds to an age-, sex-, and risk factor-adjusted risk increase of 47%, 47%, and 42% in total cardiovascular events, cardiovascular mortality, and all-cause mortality, respectively [36]. There is 1 recent case-control study evaluating arterial stiffness, measured by aortic PWV, in 20 patients with PsA in comparison to 20 matched healthy controls [37]. A significantly higher aortic PWV was found in patients with PsA compared with controls (mean ± SD, 8.2 ± 0.8 versus 6.8 ± 1.0 m/s, *P* < 0.001). The difference remained significant after adjusted for age, body weight, body height, heart rate, and mean aortic pressure. 

Results from conventional echocardiography in PsA are controversial. The study by Gonzalez-Juanatey et al. did not found significant subclinical cardiac abnormalities in 50 patients with PsA without clinically evident CVD or atherosclerosis risk factors when compared with 50 matched healthy controls [38]. A recent study by Atzeni et al. reported normal left ventricular wall thickness, size, mass, and systolic function using standard 2D echocardiography in 22 patients with PsA when compared with 35 healthy controls [31]. However, The study by Feld et al. found significantly longer PR interval (159.6 ± 21 ms versus 151.3 ± 26 ms) on electrocardiogram in 92 patients with PsA compared to 92 matched controls [39]. The abnormal prolongation of the PR interval was asymptomatic, requiring no additional intervention. And there was no statistical difference in the ORS interval or other atrial or ventricular conduction disturbance between patients with PsA and controls [39]. The study by Shang et al., by using both conventional echocardiography and tissue Doppler imaging, found that 65% of 94 PsA patients without established CVD had evidence of subclinical left ventricular dysfunction, significantly higher than that in 63 healthy controls [40]. Diastolic dysfunction appeared (38%) more common than systolic dysfunction (4%) and left ventricular dysfunction was more common in patients with cardiovascular risk factors than in those without [40]. Mitral valve prolapse was found in 14 out of 25 (56%) patients with PsA, whereas only 2 out of 32 (6%) psoriatic patients without arthritis had mitral valve prolapse [41]. In contrast, the study by Moya et al. found the prevalence of mitral valve prolapse in 44 patients with PsA (4.5%) similar to that in the general population [42].

## 6. Conventional Cardiovascular Risk Factors in PsA

It is apparent that patients with PsA have an increased prevalence of conventional cardiovascular risk factors [5, 6, 22, 23, 43, 44]. These risk factors contribute to not only overt but also subclinical CVD. 

### 6.1. Hypertension and Diabetes Mellitus

The prevalence of hypertension has been reported higher in patients with PsA compared with that in the general population [20–23, 34, 43] or with that in patients with psoriasis only [24]. The prevalence of diabetes mellitus was also found increased in PsA in the majority of [22, 34, 43, 45], but not in all [20, 21] of the previous studies. Tam et al. compared cardiovascular risk factors in 102 patients with PsA and 82 healthy controls [43]. After adjusted for body mass index (BMI), patients with PsA were still more likely to have hypertension and diabetes mellitus. Insulin resistance, measured by the Homeostasis Model Assessment Index, was also significantly increased in patients with PsA compared with that in controls [43]. The presence of hypertension and diabetes mellitus has been found significantly associated with abnormal IMT (defined as IMT  > 1.00 mm in the study) in univariate analysis, but the association became insignificant after adjusted for age and waist circumference [46]. The presence of hypertension was also found independently associated with subclinical left ventricular dysfunction in patients with PsA [40]. 

### 6.2. Smoking, BMI, and Obesity

The prevalence of current smoker was found not increased in several case-control studies [31, 34, 47] and in a population study [20] but was found significantly increased (10% versus 0%) in a case-control study by Tam et al. of 82 patients with PsA and 82 healthy controls [32]. The study by Husted et al. found a significantly lower prevalence (12% versus 24.4%) of current smoker in patients with PsA (*n* = 611) than in those with psoriasis only (*n* = 449) [24]. The relationship between smoking and (cardiovascular) mortality in PsA has not been examined [18]. Gladman et al. found that current smoking status was associated with an significantly increased risk of myocardial infarction with a hazard ratio of 15.89 [23]. But current smoking status did not increase the risk of hypertension or CVD in PsA [23]. Overall, currently, the role of smoking status in the increased cardiovascular risk in PsA is understudied. 

Increased BMI was found in 2 case-control studies by Kimhi et al. [34] and Tam et al. [43]. Patients with PsA were found to have a significantly higher waist hip ratio and a significantly higher prevalence of overweight, obesity, and abdominal obesity (according to the World Health Organization criteria) [43]. A high BMI and obesity have been frequently found associated with an increased risk of cardiovascular mortality and morbidity.

### 6.3. Metabolic Syndrome

 The metabolic syndrome is a cluster of traditional risk factors that include abdominal obesity, atherogenic dyslipidemia, hypertension, and insulin resistance. There are two studies investigating the prevalence of metabolic syndrome in patients with PsA [44, 48]. The metabolic syndrome was defined in both studies by the recent joint consensus criteria [49], when ≥3 of the following components were present: (1) elevated waist circumference according to population- and country-specific definitions; (2) elevated triglycerides level to ≥150 mg/dL, or therapy for elevated triglycerides; (3) reduced high-density lipoprotein (HDL) cholesterol to <40 mg/dL in men and <50 mg/dL in women; (4) elevated blood pressure, systolic pressure ≥130 mmHg, and/or diastolic pressure ≥85 mmHg, or requiring drug therapy; and (5) elevated fasting glucose to ≥100 mg/dL. Raychaudhuri et al. reported an increased prevalence (58.1%) of the metabolic syndrome in 105 patients with PsA compared to the 35.2% reported from the general population [48]. Mok et al. recently reported that the prevalence of the metabolic syndrome was significantly higher in PsA (38% of 109 patients) compared with matched controls (18% of 218 controls) or patients with rheumatoid arthritis (RA) (20% of 699 patients) or ankylosing spondylitis (AS) (11% of 122 patients) [44]. Patients with PsA were 2.44 times more likely to have the metabolic syndrome relative to patients with RA or AS. They had a higher risk of having central obesity, impaired fasting glucose, hypertriglyceridemia, and reduced HDL cholesterol level compared with patients with RA or AS [44].

### 6.4. Lipid

The data on lipid profiles in PsA are slightly controversial. The prevalence of hyperlipidemia was found higher in patients with PsA compared with that in general population [22] or healthy controls [34]. The prevalence of hypertriglyceridemia or hypercholesterolemia was found not increased in PsA [43]. Increased levels of total cholesterol [33] and triglyceride [32, 47] were found associated with subclinical atherosclerosis in patients with PsA. 

Jones et al. characterized the lipid profile in 50 patients with PsA and 50 age- and sex-matched controls and found that HDL cholesterol and its third subfraction, HDL_3_ cholesterol, were significantly reduced and the most dense subfraction of low-density lipoprotein (LDL), LDL_3_, was significantly increased in the patients with PsA [50]. Sub-analysis in 20 patients with active synovitis revealed significantly lower total cholesterol, LDL cholesterol, and HDL_3_ cholesterol in patients with PsA. Significantly lower levels of HDL cholesterol and its subfraction (both HDL_3_ cholesterol and HDL_2_ cholesterol) were also found by Skoczyñska et al. in 23 patients with PsA and 23 matched healthy controls [51]. The case-control study by Tam et al., however, found that patients with PsA had higher HDL cholesterol levels, lower total cholesterol and LDL cholesterol levels, and a lower total cholesterol/HDL cholesterol ratio [43]. 

 A few studies have investigated the level of Lp(a) lipoprotein, apolipoprotein A I and apolipoprotein B. Jones et al. found no significant differences in the levels of apolipoprotein A I or B, the ratio of apolipoprotein A I : HDL cholesterol or the ratio of apolipoprotein B : LDL cholesterol between patients with PsA and matched controls [50]. Twenty-five percent of the patients with PsA had raised Lp(a) lipoprotein levels (>300 mg/L) compared with 19% of controls, but the difference was not statistically significant. In contrast, Tam et al. found significantly increases in serum levels of apolipoprotein A I and apolipoprotein B and the ratio of apolipoprotein B: apolipoprotein A I in patients with PsA [43]. Oliviero et al. investigated apolipoprotein A I and total cholesterol in serum and synovial fluid in 14 patients with PsA and found that there was no statistically significant difference between patients and controls [52]. 

## 7. Nonconventional Cardiovascular Risk Factors in PsA

The increased cardiovascular risk in patients with PsA may be related to the inflammatory process. Inflammation participates centrally in all stages of the development of atherosclerosis, from the initial lesion to the end-stage thrombotic complications [53]. The increased levels of proinflammatory cytokines which are characteristics of chronic inflammatory diseases such as PsA can elicit a systemic inflammatory state that could, over time, promote atherosclerosis conducive to increased cardiovascular risk. C-reactive protein (CRP) is emerging as one of the predictors of CVD [54] and its levels also relate well with joint inflammation [55]. Patients with PsA have significantly higher level of CRP, as well as ESR, another common maker of inflammation [28, 34, 47]. In a mortality study in 428 patients with PsA by Gladman et al, high ESR (>15 mm/1st hour) was independently associated with increased overall mortality [18]. Levels of CRP and ESR were also found associated with subclinical atherosclerosis (measured by carotid IMT) [34] or endothelial dysfunction (measured by FMD%) [28] in patients with PsA. Tam et al. reported that low-grade inflammation as measured by high sensitivity CRP (hsCRP) was associated with an increase in traditional cardiovascular risk factors including a higher BMI, waist circumference, waist hip ratio, systolic and diastolic blood pressure, higher sugar level, insulin resistance, and dyslipidaemia (lower HDL cholesterol and apolipoprotein A I levels, higher total cholesterol/HDL ratio, and the ratio of apolipoprotein B: apolipoprotein A I) [43]. Higher hsCRP level was also associated with an increased thrombotic tendency reflected by increased platelet count [43]. 

The increased prevalence of obesity in patients with PsA may also increase the burden of inflammation [7]. Adipose tissue cells secret cytokines, chemokines, and hormone-like proteins which are involved in the adipose tissue homeostatis, in the regulation of insulin sensitivity and metabolism, and also in the chronic inflammation [56]. It has been found that high levels of proinflammatory cytokines, such as tumor necrosis factor alpha (TNF*α*) and interleukin-6, could down-regulate the production of adiponectin and, on the other hand, upregulate the production of leptin, which has a proinflammatory and pro-angiogenic role and can accelerate endothelial dysfunction in patients with inflammatory diseases [57]. 

Nitric oxide (NO) is the elusive mediator that causes vascular dilatation. Endothelial dysfunction occurs when the biological functions of NO are impaired [58]. Asymmetric dimethylarginine (ADMA) is a competitive inhibitor of NO synthesis [59]. Elevated levels of ADMA have been identified as a risk marker for cardiovascular disease and mortality in populations with high, intermediate, and low cardiovascular risk [60–62]. Plasma ADMA level was found significantly higher in a small sample of 22 patients with PsA compared with that in 35 healthy controls [31]. The level of ADMA was negatively associated with coronary flow reserve, a diagnostic marker for coronary artery disease, but not associated with carotid IMT or disease activity or severity [31]. 

Fibrinogen is another acute-phase protein associated with cardiovascular events [63]. The level of fibrinogen was found increased in patients with psoriasis and PsA [6] and correlated with subclinical atherosclerosis (measured by carotid IMT) [34]. Hyperhomocysteinemia confers an independent cardiovascular risk similar to that of smoking or hyperlipidemia [64]. In a study of a small number of patients with inflammatory arthritis, including PsA and RA, 10 out of 15 (67%) patients had high level of homocysteine (>15 *μ*mol/L) [65]. Asymptomatic hyperuricemia and serum uric acid level were also found correlated with subclinical atherosclerosis in patients with PsA without clinically evident CVD [66]. 

In conclusion, PsA is an inflammatory arthritis associated with increased risk for both overt and subclinical CVD. Conventional risk factors probably occur more frequently in patients with PsA; however, they only account partially for the excess cardiovascular risk. Inflammation appears as an important feature explaining the increased cardiovascular risk in PsA, by directly participating in the development of atherosclerosis and/or accentuating the established conventional cardiovascular risk factors.

## 8. Subtypes of PsA and Cardiovascular Risk

The 3 studies performed by the same group of Gonzalez-Juanatey et al. subanalyzed the influence of the pattern of arthritis of PsA on subclinical CVD, including subclinical atherosclerosis (measured by carotid IMT) [33], endothelial dysfunction (measured by FMD%) [28], and echocardiographic and Doppler abnormalities [38]. Comparisons were made between patients with polyarticular pattern and the remaining patients with PsA. There was a lack of significant differences in carotid IMT and FMD% between the two groups. There was also a lack of significant difference in echocardiographic and Doppler findings, except that patients with polyarticular pattern had smaller mean aortic root diameter (28.8 ± 4.3 mm versus 32.5 ± 5.4 mm, *P* value = 0.02) than in the rest of patients, probably due to the predominance of women in the group of polyarticular arthritis (58% versus 24%). The relationship between the pattern of arthritis and abnormal carotid IMT (defined as IMT > 1.00 mm) was studied by Mazlan et al. on 63 patients with PsA [46]. The percentage of abnormal IMT did not differ significantly between patients with polyarticular and those with nonpolyarticular disease, between patients with oligoarticular and those with nonoligoarticular disease, and between patients with and those without spondylitis. Overall, the pattern of arthritis appears to have limited influence on cardiovascular risk in PsA. 

## 9. Effect of Treatments on Cardiovascular Risk in PsA

### 9.1. Nonsteroidal Anti-Inflammatory Drugs (NSAIDs) and Disease-Modifying Antirheumatic Drugs (DMARDs)

There is evidence suggesting that NSAIDs including selective cyclo-oxygenase-2 (COX2) inhibitors and traditional NSAIDs are associated with increased risk of CVD. In a meta-analysis of 138 randomized trials, selective COX2 inhibitors were associated with a moderate increase (43%) in the risk of severe cardiovascular events, mostly attributable to an increased risk of myocardial infarction (odds ratio 1.86, 95% confidence interval 1.33–2.59) [67]. High-dose ibuprofen and diclofenac, but not high-dose naproxen, were also associated with excess cardiovascular events. However, there is no study that specifically assesses the cardiovascular risk related to NSAIDs in patients with PsA. The net effects of NSAIDs and COX2 inhibitors in patients with inflammatory arthritis are difficult to determine, because on one hand they increase the cardiovascular risk, but on the other hand they decrease the risk due to their favorable effect on controlling the disease. In a mortality study of PsA, the number of medications prior to the study was positively associated with overall mortality rate [18]. However, increased mortality associated with medications in PsA could not be fully established since the number of medications could also be a surrogate indicator of the severity of the disease, which is associated with mortality [18]. Use of DMARDs, including methotrexate, sulphasalazine, and leflunomide, was not found associated with abnormal carotid IMT (defined as IMT  > 1.00 mm in the study) [46]. 

### 9.2. Biologic DMARDs

Previous findings support the pivotal role of inflammation in atherogenesis. However, till now, evidence is not uniform and adequate studies are lacking to support that aggressive anti-inflammation therapy, including biologic DMARDs, lowers the cardiovascular risk in patients with PsA. 

Results of the effect of infliximab on lipid profile are controversial. A reduction in HDL cholesterol and an elevation in total triglyceride, with no changes in total cholesterol and LDL cholesterol, were reported in a 6-week prospective study of 8 patients with PsA treated with infliximab [68]. A recent 24-week study of 15 patients with PsA treated with infliximab also found significantly increased total triglyceride levels, along with increased very-low-density lipoprotein cholesterol level, but with no changes in total cholesterol, LDL cholesterol, and HDL cholesterol levels [69]. In contrast, a sustain increase in HDL with no significant changes in other lipids was reported in a 24-week study of 24 patients with PsA treated with infliximab [70]. Significant weight gain was observed in treatment of infliximab and etanercept [71]. 

A 12-week randomized double-blind, placebo-controlled clinical trial was conducted to examine the effect of a TNF*α* blocker, onercept, on cardiovascular risk factors in 127 patients with PsA [72]. Onercept at a dose of 100 mg induced significant reductions in the levels of CRP, Lp(a) lipoprotein, and homocysteine and an increase in the levels of sex hormone binding globulin (as a surrogate marker of insulin resistance) and apolipoprotein A I. But the level of circulating adiponectin, an anti-inflammatory and potentially antiatherogenic molecule, did not change significantly over time [73]. These data support an important precursor role for high-grade inflammation in modulating the putative risk parameters. However, the results also indicated that treatment of onercept increased levels of triglyceride and apolipoprotein B, which suggested that it was not possible to prove the cardioprotective effect of anti-TNF*α* therapies on the basis of biochemical changes in isolation, and direct measures of atherosclerotic progression such as carotid ultrasonography would be preferred. 

In a cross-sectional study, carotid IMT was higher in 104 patients with PsA on synthetic DMARDs than in those on anti-TNF*α* therapy (120 patients) [74]. In a pilot prospective study, Tam et al. showed that 12-week anti-TNF*α* therapy may be associated with significant reduction in carotid IMT, along with improvement in clinical and laboratory manifestations in patients with PsA [75]. The 2-year analysis showed that further regression of the maximum IMT was possible only in patients who were continued on long-term anti-TNF*α* therapy, suggesting that effective suppression of inflammation in patients with high-grade inflammation may potentially reverse early atherosclerotic lesions. Anti-TNF*α* therapies were also shown to improve aortic stiffness [76, 77] and modify the endothelial function [78] in small samples of patients with inflammatory arthritis, including PsA. 

## 10. Recommendations for Cardiovascular Risk Management

The European League Against Rheumatism (EULAR) has developed recommendations for cardiovascular risk management in patients with RA and other inflammatory arthritis, including AS and PsA [79]. The EULAR recommendations recognize the association between inflammation and atherosclerosis in patients with inflammatory arthritis and recommend that aggressive suppression of disease activity or inflammation is necessary to lower the cardiovascular risk. Annual assessment of cardiovascular risk using national guidelines is recommended for all patients with PsA. Any risk factors identified should be managed according to local guidelines. In the absence of local guidelines, cardiovascular risk management should follow the Systematic COronary Risk Evaluation (SCORE) model. Unlike the Framingham risk score, in which the pragmatic outcome bases on both cardiovascular mortality and morbidity [80], the SCORE model estimates the 10-year risk of developing cardiovascular death and includes the following risk factors: age, gender, smoking habit, systolic blood pressure, and either total cholesterol or the total cholesterol/HDL cholesterol ratio [81]. Statins, angiotensin-converting enzyme inhibitors, and/or angiotensin II blockers are preferred treatment options due to their potential anti-inflammatory effects. Prescribing COX2 inhibitors and most NSAIDs in patients with a documented CVD or in the presence of cardiovascular risk factors should be cautious due to their potential cardiovascular risk.

## 11. Conclusion

PsA is associated with an increased risk of clinical and subclinical CVD mostly due to accelerated atherosclerosis. Chronic inflammation plays a pivotal role in the pathogenesis of atherosclerosis in PsA, acting independently and/or synergistically with the traditional risk factors. Large-scale prospective study is in need to identify risk factors of CVD in PsA. There is a need for large long-term interventional trials with cardiovascular end points to investigate whether benefits in articular disease achieved by aggressive suppression of inflammation translate into reduced cardiovascular risk in PsA. 

## Figures and Tables

**Table 1:** Mortality studies in psoriatic arthritis.

Study	Year	Country	no. of patients	Male, no. [%]	Follow-up period	SMR (95% CI)	Remarks	Major causes of death
Roberts et al. [11]	1976	UK	168	—	—	—	18 deaths occurred	9 deaths due to CVD
Coulton et al. [13]	1989	UK	40	16 (40)	8 years	—	No deaths occurred	—
Wong et al. [8]	1997	Canada	428	234 (54.7)	1978 –1993	Overall: 1.62 (1.21–2.12) Male: 1.65 (1.09, 2.04) Female: 1.59 (1.04, 2.33)	—	CVD (36.2%), respiratory disease (21.3%)
Shbeeb et al. [10]	2000	US	66	32 (48.5)	1982–1991	—	Similar survival to the general population	—
McHugh et al. [3]	2003	UK	87	38 (57.6)	Median: 65 months	—	9 deaths occurred	5 deaths due to CVD
Alamanos et al. [16]	2003	North-west Greece	221	108 (48.9)	1982–2001	—	4 deaths occurred	2 deaths due to CVD
Ali et al. [15]	2007	Canada	680	385 (56.6)	1978–2004	Overall: 1.36 (1.12–1.64) Male: 1.25 (0.95, 1.65) Female: 1.47 (1.13, 1.91)	—	Malignancy (23.6%), CVD (24.5%), respiratory diseases (10.4%)
Wilson et al. [9]	2009	US	147	90 (61)	1970–1999	0.91 (0.58, 1.37)	—	—
Buckley et al. [14]	2010	UK	453	232 (51.2)	1985–2007	Overall: 0.82 (0.58–1.13) Male: 0.68 (0.39, 1.10) Female: 0.97 (0.60, 1.48)	—	CVD (38%), respiratory disease (27%), malignancy (14%)
Ahlehoff et al. [17]	2010	Denmark	607	—	1997–2006	—	RR for all-cause mortality*: 1.74 (1.32–2.30); RR for cardiovascular death: 1.84 (1.11–3.06)	—
Mok et al. [12]	2011	Hong Kong	778	424 (54)	1999–2008	Overall: 2.50 (1.81–3.19) Male: 2.27 (1.44, 3.10) Female: 2.76 (1.61, 3.91)	—	Infection (33%), malignancy (20%), CVD (20%)

SMR: standardized mortality ratio; CI: confidence interval; UK: United Kingdom; CVD: cardiovascular disease; US: United States; RR: relative risk.

*Rate ratio (95% confidence interval) compared with 4,003,265 controls, adjusted for age, calendar year, concomitant medication, comorbidity, socio-economic data and gender.

**Table 2:** Studies on subclinical atherosclerosis in psoriatic arthritis.

	Year	Country	No. of patients	No. of controls	Male, no.	Age, mean ± SD	IMT, mm, PsA	IMT, mm, controls	*P* value	Carotid plagues, no. (%), PsA	Carotid plagues, no. (%), controls	Carotid plagues, *P* value
Gonzalez-Juanatey et al. [33]	2007	Spain	59	59	31	48.8 ± 12.4	0.699 ± 0.165	0.643 ± 0.111	0.031	9 (15)	3 (5)	0.068
Kimhi et al. [34]	2007	Israel	47	100	24	50 ± 13.5	Average IMT: 0.76 ± 0.11; adjusted IMT*: 0.726 ± 0.075	Average IMT: 0.64 ± 0.27; adjusted IMT*: 0.689 ± 0.077;	Average IMT: <0.0001; adjusted IMT*: 0.04	—	—	—
Eder et al. [47]	2008	Israel	40	40	12	57.85 (43–76)^†^	1.04 ± 0.35	0.88 ± 0.28	0.03	28 (70)	16 (40)	<0.05
Tam et al. [32]	2008	Hong Kong	82	82	42	49 ± 10	Average IMT: 0.74 ± 0.126; maximum IMT: 0.888 ± 0.178	Average IMT: 0.626 ± 0.074; maximum IMT: 0.722 ± 0.106	Average IMT: <0.001; maximum IMT: <0.001	15 (18)	0	<0.001
Mazlan et al. [46]	2009	Malaysia	63	—	28	49.92 (13–74)^†^	0.725 ± 0.260	—	—	—	—	—
Atzeni et al. [31]	2011	Italy	22	35	12	54.9 ± 12.97	0.64 ± 0.26	0.62 ± 0.52	NS	—	—	—

IMT: intima-media thickness; PsA: psoriatic arthritis; NS: not significant.

*Adjusted for age, hyperlipidemia, diabetes, and body mass index.

^†^Mean (range).
